# Subjective cognitive decline detected by the oscillatory connectivity in the default mode network: a magnetoencephalographic study

**DOI:** 10.18632/aging.102859

**Published:** 2020-02-25

**Authors:** Chia-Hsiung Cheng, Pei-Ning Wang, Hui-Fen Mao, Fu-Jung Hsiao

**Affiliations:** 1 Department of Occupational Therapy and Graduate Institute of Behavioral Sciences, Chang Gung University, Taoyuan, Taiwan; 2 Healthy Aging Research Center, Chang Gung University, Taoyuan, Taiwan; 3 Department of Psychiatry, Chang Gung Memorial Hospital, Linkou, Taiwan; 4 Laboratory of Brain Imaging and Neural Dynamics (BIND Lab), Chang Gung University, Taoyuan, Taiwan; 5 Brain Research Center, National Yang-Ming University, Taipei, Taiwan; 6 Division of General Neurology, Department of Neurological Institute, Taipei Veterans General Hospital, Taipei, Taiwan; 7 Department of Neurology, National Yang-Ming University, Taipei, Taiwan; 8 School of Occupational Therapy, College of Medicine, National Taiwan University, Taipei, Taiwan; 9 Department of Physical Medicine and Rehabilitation, National Taiwan University Hospital, Taipei, Taiwan

**Keywords:** subjective memory complaint, functional connectivity, resting state, delta oscillation, gamma oscillation

## Abstract

Discriminating between those with and without subjective cognitive decline (SCD) in cross-sectional investigations using neuropsychological tests is challenging. The available magnetoencephalographic (MEG) studies have demonstrated altered alpha-band spectral power and functional connectivity in those with SCD. However, whether the functional connectivity in other frequencies and brain networks, particularly the default mode network (DMN), exhibits abnormalities in SCD remains poorly understood. We recruited 26 healthy controls (HC) without SCD and 27 individuals with SCD to perform resting-state MEG recordings. The power of each frequency band and functional connectivity within the DMN were compared between these two groups. Posterior cingulate cortex (PCC)-based connectivity was also used to test its diagnostic accuracy as a predictor of SCD. There were no significant between-group differences of spectral power in the regional nodes. However, compared with HC, those with SCD demonstrated increased delta-band and gamma-band functional connectivity within the DMN. Moreover, node strength in the PCC exhibited a good discrimination ability at both delta and gamma frequencies. Our data suggest that the node strength of delta and gamma frequencies in the PCC may be a good neurophysiological marker in the discrimination of individuals with SCD from those without SCD.

## INTRODUCTION

Although mild cognitive impairment (MCI) has been accepted as a symptomatic pre-dementia stage, over the past decade there has been an increasing interest in the pre-clinical asymptomatic stage of dementia, i.e., subjective cognitive decline (SCD). According to the definition provided by the Subjective Cognitive Decline Initiative Working Group, SCD refers to a self-perceived worsening in cognitive capacity but a normal age- and education-adjusted performance on standardized cognitive assessments, which are used to diagnose MCI and dementia [1]. It has been documented that SCD is potentially a pre-MCI stage. Support for this contention is derived from longitudinal cohort studies showing strong associations between SCD and prospective objective cognitive performance, as well as the incidence of dementia [2–5]. Despite these promising findings, it remains difficult to investigate the functional correlates of SCD through cross-sectional studies. This is because the detection of subtle cognitive changes using neuropsychological assessments at one particular time point is challenging [6, 7]. However, the cross-sectional identification of neural signatures of SCD has a great clinical impact on the early detection and prognostic prediction of the dementia spectrum.

Individuals with SCD can be distinguished from healthy older individuals without SCD in cross-sectional studies using functional imaging technologies. Previous functional magnetic resonance imaging (MRI) research has revealed a reduction of functional connectivity in individuals with SCD versus healthy controls (HC) [8–10]. However, other studies have shown an opposite pattern, indicating increased functional connectivity in SCD [11, 12]. Compared with hemodynamic responses, electrophysiological recordings, such as electroencephalography (EEG) and magnetoencephalography (MEG), are able to directly measure the neural activities in the time and/or frequency domains [13, 14]. For example, available resting EEG studies have shown elevated alpha [15] and delta [16] powers in SCD versus HC. However, a recent MEG study showed a reduced alpha power in bilateral prefrontal and occipital lobes [17]. In addition, Lopez-Sanz et al. found a disruption of alpha-band functional connectivity in SCD [18]. In the aforementioned findings, the power spectral characteristics in different frequency bands are inconsistent. Furthermore, apart from the alpha band, whether the functional connectivity in other frequencies and brain networks exhibits abnormalities in SCD remains poorly understood.

In the research of resting-state brain activities, the default mode network (DMN) is a key network associated with episodic memory function; it is affected in patients with MCI [19, 20] and dementia [21, 22]. The DMN is composed of several cortical hubs, including the posterior cingulate cortex (PCC), precuneus (PCu), lateral temporal cortex (LTC), medial temporal cortex, medial frontal cortex, and inferior parietal lobule [23–25]. Among the DMN nodes, PCC is considered a key region because it is the only hub that directly interacts with other DMN nodes [26]. Previous functional MRI or positron emission tomography (PET) studies have demonstrated a disruption of DMN with PCC as a node in patients with MCI and Alzheimer’s disease (AD) [27–29]. There is also evidence demonstrating that, compared with younger adults, activation of the PCC in the elderly shows a significant reduction; this reduction is markedly greater in patients with dementia [30]. These findings suggest that the PCC plays a vital role in differentiating dementia from healthy aging. However, utilization of the spectral power of the PCC or the characteristics of functional connectivity with the PCC for the segregation of SCD from HC remains undocumented.

MEG, as it possesses a better spatial resolution than EEG, is suitable for elucidating the local cortical activities and functional connectivity in different oscillatory bands at the source level. Moreover, the minimum norm estimate (MNE) is a distributed source imaging method that can reconstruct a number of distributed neural generators overlapping in time [31]. Hence, MNE is considered as a preferred method when investigating multiple sources compared with other reconstruction solutions [32, 33].

Specifically, the present study had three aims. Firstly, at the regional level, we attempted to comprehensively examine whether the resting-state power of delta, theta, alpha, beta, and gamma oscillations in the DMN nodes would show significant differences between individuals with and without SCD. Secondly, at the network level, we sought to investigate whether individuals with SCD would show aberrant connectivity among the DMN nodes at specific frequency bands. Finally, given the crucial role of the PCC in the DMN, we aimed to test whether PCC-based connectivity strength could serve as a good neurophysiological marker for the discrimination of individuals with SCD from HC.

## RESULTS

### Demographics and clinical profiles

Table 1 shows the demographic data and neuropsychological assessment scores of the HC and SCD groups. The two groups did not significantly differ in terms of gender distribution, age, living status, or years of education. In addition, both groups exhibited equivalent cognitive performance except for in the Boston Naming Test, in which the score of phonemic cues of the SCD group was significantly lower than that of the HC group. The frequencies of apolipoprotein E ε4 (APOE 4) carriers also did not significantly differ between these two groups.

**Table 1:** Demographic variables and neuropsychological measures as mean ± SD.

	**HC (n = 26)**	**SCD (n = 27)**	**p**
Sex (male/female)	9/17	9/18	0.92
Age (years)	67.00 ± 8.10	67.00 ± 9.29	1.00
Education (years)	13.27 ± 2.97	12.85 ± 3.28	0.63
Living status (alone/not alone)	3/23	4/23	0.73
APOE 4 (yes/no)	6/20	6/21	0.94
MMSE	28.96 ± 0.96	29.11 ± 1.09	0.60
STM	2.58 ± 0.70	2.52 ± 0.80	0.78
CVVLT			
Total	31.19 ± 3.26	31.44 ± 3.84	0.80
Delayed	8.38 ± 0.85	8.59 ± 0.64	0.32
WMS Logic memory			
Immediate	15.31 ± 3.73	16.11 ± 3.72	0.44
Delayed	14.38 ± 4.02	14.63 ± 3.76	0.82
CFT			
Copy	32.62 ± 2.38	32.93 ± 2.18	0.62
Immediate	25.37 ± 6.16	24.30 ± 7.24	0.57
Delayed	24.73 ± 6.44	23.54 ± 7.02	0.52
VFT- animal	19.27 ± 4.41	18.41 ± 5.21	0.52
BNT			
Spontaneous	27.00 ± 2.37	27.56 ± 2.01	0.36
Semantic cues	0.38 ± 0.57	0.41 ± 0.64	0.89
Phonemic cues	1.81 ± 1.41	0.96 ± 0.90	0.01
Digit Span Test			
Forwards	8.35 ± 1.16	8.44 ± 0.70	0.71
Backwards	5.50 ± 1.56	5.67 ± 1.36	0.68
Trail Making Test			
Part A (s)	17.19 ± 15.88	13.48 ± 10.74	0.32
Part B (s)	37.62 ± 26.42	37.22 ± 21.96	0.95

SD, standard deviation; HC, healthy control; SCD, subjective cognitive decline; STM, short-term memory; CVVLT-total, Chinese-version Verbal Learning Test-total immediate recall; CVVLT-delayed, Chinese-version Verbal Learning Test-long delayed free recall (10 min); WMS-Logic memory, Wechsler Memory Scale-Logic memory; CFT, Rey-Osterrieth Complex Figure Test; VFT-animal, Verbal Fluency Test-animal; BNT, Boston Naming Test.

### Resting-state spectral power in the DMN

The patterns (peak power frequency and power distribution) of the resting-state oscillatory activities in each region of interest (ROI) were comparable between the HC and SCD groups. Peak frequency shifting or abnormal power increases were not observed in the SCD group. Accordingly, statistical examination of the normalized spectral power values of each ROI at each frequency band did not yield a significant main effect for groups (all p > 0.05). This finding indicated that the normalized powers of each frequency band in the identified ROIs were not conspicuously altered in SCD.

### Resting-state functional connectivity in the DMN

**Figure 1:**
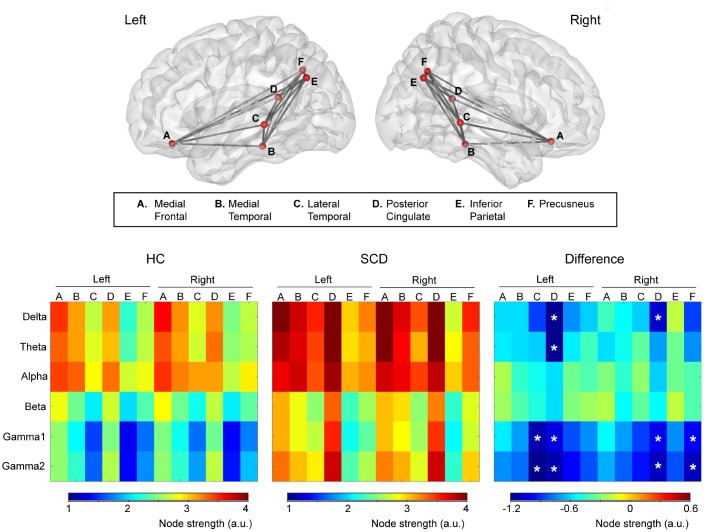
**Upper panel**: The intrinsic functional connectivity of default mode network viewed from the left and right side. **Lower panel**: Node strength of each brain area from delta to gamma2 bands was color-coded for healthy controls (HC) and individuals with subjective cognitive decline (SCD). The difference (HC minus SCD) of the node strength demonstrated the alterations of functional connectivity due to SCD. *p < 0.05.

The node strength of each ROI within the DMN is shown using color-coded values for the delta to gamma2 bands in the HC and SCD groups (Figure 1). The difference map (HC minus SCD) of node strength demonstrated the increased DMN connectivity at specific bands and regions for the SCD group. Significant differences were observed at the delta band in the bilateral PCC (left, HC: 3.1 ± 0.9 vs. SCD: 4.4 ± 1.6; right, HC: 3.2 ± 1.0 vs. SCD: 4.3 ± 1.6); at the theta band in the left PCC (HC: 3.0 ± 0.8 vs. SCD: 4.2 ± 1.6); at the gamma1 and gamma2 bands in the left LTC (gamma1, HC: 1.5 ± 0.3 vs. SCD: 2.6 ± 1.7; gamma2, HC: 1.6 ± 0.4 vs. SCD: 2.9 ± 1.7), the right PCu (gamma1, HC: 1.7 ± 0.4 vs. SCD: 2.7 ± 1.5; gamma2, HC: 1.9 ± 0.5 vs. SCD: 3.0 ± 1.6) and the left (gamma1, HC: 2.4 ± 0.9 vs. SCD: 3.5 ± 1.4; gamma2, HC: 2.6 ± 1.0 vs. SCD: 3.7 ± 1.5), and the right PCC (gamma1, HC: 2.4 ± 1.0 vs. SCD: 3.4 ± 1.4; gamma2, HC: 2.6 ± 1.1 vs. SCD: 3.7 ± 1.4) (all corrected p < 0.05). In contrast, there was no clear difference in DMN connectivity at the alpha and beta bands (all p > 0.05).

**Figure 2:**
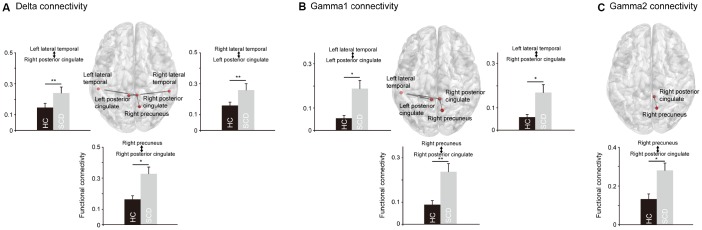
**The significant differences regarding the functional connectivity between healthy controls (HC) and individuals with subjective cognitive decline (SCD).** In the delta (**A**) and gamma1 (**B**) oscillations, SCD showed significantly stronger functional connectivity between posterior cingulate cortex and precuneus, and between posterior cingulate cortex and lateral temporal cortex. In the gamma2 oscillations (**C**), SCD showed significantly stronger functional connectivity between posterior cingulate cortex and precuneus. *p < 0.05, **p < 0.01.

The cortical areas with significant between-group differences were further examined in terms of functional connectivity in DMN to detect the altered connections in SCD (Figure 2). At the delta band, amplitude envelope correlation (AEC) values in SCD were increased in the connectivity between the left LTC and right PCC (p = 0.0005), right LTC and left PCC (p = 0.0005), and right PCu and right PCC (p = 0.0005). At the gamma1 band, increased functional connectivity in SCD was observed between the left LTC and left PCC (p = 0.0012), left LTC and right PCC (p = 0.0044), and right PCu and right PCC (p = 0.0007), as well as at the gamma2 band in the connectivity between right PCu and right PCC (p = 0.0023). In general, for the resting-state neural activities of the PCC within the DMN, the alterations correlated with the individuals with SCD were increases in node strength and functional connectivity.

### Network measurement for the detection of SCD

**Figure 3:**
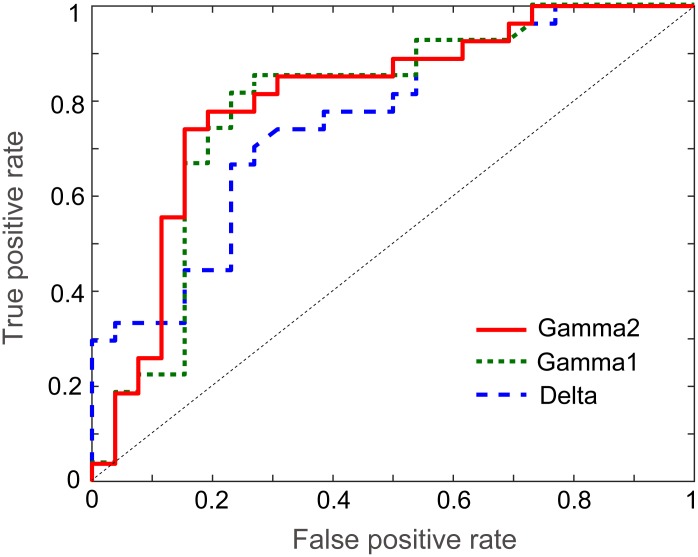
**Receiver operating characteristic curves of delta, gamma1 and gamma2 frequency bands for the discrimination of healthy controls from individuals with subjective cognitive decline.**

Receiver operating characteristic (ROC) curve analysis was used to evaluate whether network measurement could serve as a good neurophysiological marker for the discrimination of SCD from HC. In this study, the node strength and AEC values with significant group differences were used in the ROC curve analysis; moreover, the summation of node strength from bilateral ROIs was also explored. Notably, node strength in the PCC exhibited a good discrimination ability at the delta (area under curve [AUC] = 0.759, sensitivity = 0.704, specificity = 0.731, p = 0.001), gamma1 (AUC = 0.789, sensitivity = 0.851, specificity = 0.769, p < 0.001) and gamma2 (AUC = 0.803, sensitivity = 0.778, specificity = 0.808, p < 0.001) bands (Figure 3). In contrast, when using node strength or AEC values in other DMN areas, all the AUC values were < 0.7 at the corresponding bands.

Based on these findings, we further examined whether higher node strength correlated with more cognitive complaints. Our results showed that the node strength of the PCC at the gamma1 band was significantly associated with more complaints of cognitive decline among individuals with SCD (rho = 0.252, p = 0.035, one-tailed).

## DISCUSSION

The goal of this cross-sectional study was to investigate the functional correlates, both at the regional and network levels, underlying SCD by means of resting-state MEG imaging. There were no significant differences observed between groups regarding the peak frequency and power in each identified DMN node. However, compared with the HC group, the SCD group showed significantly increased delta-band connectivity between the LTC and PCC, and between the PCu and PCC. In addition, the SCD group exhibited elevated gamma-band connectivity between the LTC and PCC, and between the PCu and PCC. The ROC curve data also showed that the node strength of the delta and gamma frequencies in PCC was a good neurophysiological marker for the discrimination of SCD from HC.

At the regional level, we did not find significant between-group differences in the spectral characteristics at each frequency band. The slowing of alpha peak frequency has been well documented in patients with MCI [17, 34–36] and AD [35, 37, 38]. However, investigation of alpha frequencies in SCD has been limited. The first study, conducted by Alexander et al. (2006), did not detect the slowing of peak alpha frequency between those with and without SCD [15]. A recent study performed by Lopez-Sanz et al. (2016) also showed comparable peak alpha frequency values between SCD and HC [17], in line with our present MEG data. In the comparisons of power between HC and SCD, the results were extremely contentious. By calculating the absolute power values, Alexander et al. found an elevated alpha strength in SCD, which significantly correlated with better performance in the Digit Span Backward Test; on the other hand, by using a relative power method, a significantly decreased alpha power over wide brain regions was observed in SCD versus HC [15]. In contrast to the aforementioned findings, we did not detect any significant difference in relative power at any of the frequency bands, including alpha, between the HC and SCD groups using MEG combined with MNE source modeling. Taken together, although SCD is likely a pre-MCI stage, the available empirical results suggest that regional spectral characteristics (in either frequency or power) do not have sufficient capacity to separate SCD from HC through cross-sectional examination.

A long-held viewpoint has suggested the DMN, at the macroscopic scale, to be a key network that can reflect pathological changes across the spectrum of dementia [39, 40]. In particular, the PCC is recognized as a critical node in the DMN [41]. A previous functional MRI study revealed increased functional connectivity in the DMN (including the PCC, left PCu, right hippocampus, and right superior temporal gyrus) in SCD versus HC [11]. Our present MEG study also highlighted the role of the PCC in DMN functional connectivity, revealing that individuals with SCD demonstrated significantly increased delta- and gamma-band connectivity between the PCC and LTC, and between the PCC and PCu, compared with HC. The exact neural mechanism underlying the increased delta- and gamma-band functional connectivity in SCD remains poorly understood. A plausible, but at this stage likely speculative explanation, may be that the increased PCC functional connectivity reflects a compensation mechanism in the very early stages of memory impairments. The enhanced connectivity may compensate for inefficient processing elsewhere in the brain, which was not explored in the present study. However, a longitudinal study is warranted to follow up with these participants with SCD and compare those who remain stable with those who progress to MCI or AD. Another tentative suggestion is that the increased PCC functional connectivity may be related to pathological changes [42, 43] or temporary adaptations to the subjective memory complaints, which exhibit an upregulation of synchronization in the asymptomatic SCD stage followed by a breakdown of the functional connectivity in the MCI or AD stage.

Although delta rhythm has been well documented to be involved in the integration of cerebral activity with homeostatic functions, such as reward and autonomic and metabolic processes, an increasing body of evidence also suggests that it is implicated in many aspects of cognitive processing, including attention and memory [44]. For example, an increment of baseline power in the delta frequency band has been found in patients with AD [45, 46]. In the present study, despite the absence of significant between-group differences in delta power in the regional DMN nodes, we hypothesized that SCD, a very subtle symptom on the neurodegenerative spectrum, may still exhibit abnormalities. These abnormalities are initially in the form of functional connectivity and subsequently in the form of local dysfunction after symptom progression. In addition, delta activity is sensitive to internal stimuli signaling, such as pain, fatigue, and hypoxia [44]. Extending the available findings, we further suggest that the increased delta-band functional connectivity observed in SCD may also be related to the sensitivity of internal cognitive ability. Notably, all participants with SCD in this study proactively sought medical help due to worries and sensitivities regarding their self-reported cognitive deterioration. Further studies comparing participants with SCD both with and without worries/sensitivities are warranted to validate this hypothesis.

One may argue that the gamma oscillations pertain to a relatively local, rather than long-range, synchronization. Compared with the HC group, we found that the SCD group demonstrated stronger gamma oscillations over short-distance (e.g., right PCC to right PCu) and long-distance (e.g., right PCC to left LTC) connectivity. Over the past decade, there has been increasing evidence suggesting that gamma-band oscillations are involved in both local and long-range connectivity [47]. For example, Rodriguez-Rojo et al., recruiting 36 cognitively intact older females, found that compared to BDNF Val/Val individuals, those with Val/Met showed diminished antero-posterior gamma connectivity (e.g., left gyrus rectus-right lateral superior occipital lobe) [48]. Although the mechanisms behind the increased gamma-band connectivity in SCD versus HC remain elusive, our present results suggest that the altered synchronization of this frequency band in both short- and long-distance connectivity may represent one of the underlying neural signatures of SCD.

To the best of our knowledge, this was the first study utilizing ROC curve analysis to determine the diagnostic values of these connectivity patterns in SCD based on the significant between-group differences in the node strength and AEC values. Node strength in the PCC showed good accuracy in discriminating SCD from HC, suggesting that the PCC may also play a critical role in the early identification of SCD at the individual level. Previous empirical studies have shown that the metabolism and regional blood flow of the PCC is potentially one of the earliest markers to predict cognitive deterioration from MCI to AD [49–51]. With a seed in the PCC, functional connectivity appeared to be further disrupted with the increasing severity of AD [52]. It is valuable for future research to use PCC functional connectivity in the characterization of the different stages of cognitive impairments, from asymptomatic SCD to severely symptomatic dementia.

There were a few limitations in the present study. Firstly, the results were limited by a relatively modest sample size. However, based on our literature review, sample sizes with >20 participants in each group have been considered reasonable and statistically powerful [53, 54]. Secondly, the current study pertained to an exploratory research design. Based on our results, additional longitudinal studies are warranted to further verify the role of PCC functional connectivity in predicting the development of objective cognitive decline and disease progression. Thirdly, the spatial resolution and localization accuracy of MEG is considered limited in the deep brain structures, such as midline cortical regions. However, there is increasing evidence showing that the posterior midline cortices (e.g., PCu and PCC) could be reliably localized using MEG recordings [55, 56]. More importantly, a recent resting-state MEG study demonstrated that the reconstruction of posterior midline cortical activation by MNE, which is the method we applied in the present study, was markedly better than that obtained by the linearly constrained minimum variance Beamformer [57]. Nevertheless, cautions should be exercised in relating the present findings to those of functional MRI studies. Finally, up to the date, there is no consensus on the assessment of SCD. Although there have been some self-reported and informant-reported questionnaires with psychometric validation [58], their clinical utility or application in the general population requires further improvement. For example, the Everyday Cognition scale exhibits excellent discrimination ability between those with and without dementia [59, 60]. However, in this questionnaire, the participants are asked to rate their ability to perform certain everyday tasks at present versus 10 years earlier [59, 60]. When applying this assessment to individuals with SCD, it may not be feasible for them to compare their cognitive function using such a long-term timeframe. Thus, future studies on SCD are warranted to develop clinically feasible assessments.

In conclusion, our MEG data demonstrated an increased DMN functional connectivity in individuals with SCD versus HC, suggesting that the self-reported subjective cognitive complaint is a reflection of objective alterations in brain function. Furthermore, the node strength of the delta and gamma frequencies in the PCC may be a good imaging indicator in the discrimination of SCD from HC.

## MATERIALS AND METHODS

### Research participants

A total of 27 individuals with SCD (nine males; mean age: 67 ± 9.3 years) were recruited from the memory clinic of the Department of Neurology, Taipei Veterans General Hospital (Taipei, Taiwan). All participants with SCD were seeking help for self-experienced cognitive decline compared with their level two years earlier. They had to report at least one complaint by responding to 12-item questionnaire such as: “Do you usually forget where you put objects (such as keys or glasses) in your home or office?”, “Do you have any difficulty in remembering specific facts from a newspaper or TV program after you have just finished it?”, “Do you usually forget appointments with your friends or doctor?”, “Do you have any difficulty in word-finding during conversations with others?”, etc. In addition, they exhibited normal age- and education-adjusted neuropsychological performance with the absence of a diagnosis of MCI or dementia [1]. Healthy community-dwelling older adults without reported cognitive decline (N = 26; nine males; mean age: 67 ± 8.1 years) were also recruited in the present study, forming the healthy control group (HC). None of the SCD or HC participants had a history of major neurological or psychiatry disorders, including coronary heart disease, stroke, Parkinson’s disease, depression, anxiety, etc.

None of the participants received treatment with anti-dementia drugs. In the HC group, one participant was on an antidepressant and two participants were receiving treatment with benzodiazepines. In the SCD group, some participants were receiving treatment with an antidepressant (n=1), z-drug (n=1), benzodiazepines (n=3), or a combination of benzodiazepines and z-drug (n=1). However, all participants were asked to refrain from taking their medication 24 h prior to the MEG recordings to minimize the potential effects on brain imaging data.

This study was approved by the Institutional Review Board of Taipei Veterans General Hospital, and was performed in accordance with approved guidelines and regulations. Written informed consent was provided by all participants.

### Neuropsychological assessments

All participants were Chinese and underwent the following Chinese-version neuropsychological tests: Mini-Mental State Examination, Chinese-version Verbal Learning Test, Wechsler Memory Scale-Logic Memory Test, Boston Naming Test, Rey-Osterrieth Complex Figure Test, Trail Making Test part A and B, Digit Span Forward and Backward Test, and Verbal Fluency Test. Apolipoprotein E ε4 (APOE 4) genotyping was also performed for all participants.

### MEG recordings

Neuromagnetic data were recorded using a whole-scalp 306-channel MEG (Vectorview; Elekta Neuromag, Helsinki, Finland), composed of 102 identical triple sensor elements (one magnetometer and two orthogonal planar gradiometers). Four coils, representing the head position, were placed on the scalp of the participant with their positions in the head coordinate frame. The positions were specified by the nasion and two pre-auricular points using Cartesian coordinates and measured with a three-dimensional digitizer. For accurate registration, approximately 100 additional scalp points were also digitized. These landmarks of the head position enabled further alignment of the MEG and MRI coordinate systems. Moreover, electrooculography (EOG) and electrocardiography (ECG) recordings were also simultaneously performed during the MEG recordings. Individual brain MR images were captured using a 3T MR system (Discovery 750; GE Medical Systems, Milwaukee, Wisconsin, USA), with a repetition time of 9.4 ms, echo time of 4 ms, recording matrix of 256 × 256 pixels, field of view of 256 mm, and slice thickness of 1 mm.

The MEG recordings were initiated with a 3-min empty-room recording to capture sensor and environmental noise, which was used to calculate the noise covariance for offline source analysis. In the subsequent 5-min resting-state recordings with a digitization rate of 1,000 Hz, the participants sat comfortably with their head supported by the helmet of the MEG. They were asked to close their eyes, and remain awake and relaxed. The recordings were terminated and repeated if the participant fell asleep or had excessive head movement during the recordings.

### Data preprocessing

**Figure 4:**
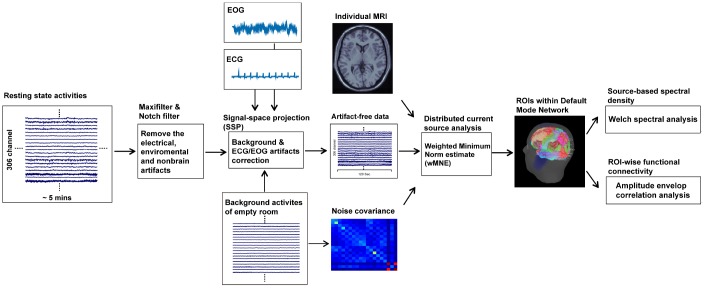
**Procedures of resting-state MEG data analysis.**

We took the following strategies to exclude the contaminations of non-brain or environmental artifacts from spontaneous MEG data: (1) we applied the MaxFilter from the Neuromag software system [61, 62]; (2) we visually inspected all data for segments containing artifacts caused by head movements or environmental noise and discarded the contaminated segments; (3) notch filters (60 Hz and its harmonics) were used to remove powerline contaminations; and (4) identified heartbeat and eye blinking events from ECG and EOG data were used to independently define the projectors through principal component analysis. The principal components meeting the artifact’s sensor topography were then manually selected and excluded through orthogonal projection [63]. Furthermore, T1-weighted structural volumetric images were automatically reconstructed into the surface model for further source analysis using BrainVISA (4.5.0, http://brainvisa.info). The detailed geometric reconstruction of the scalp, brain gray and white matter, and tessellations provided a topographical three-dimensional representation of the brain surface and was used to estimate the gray and white matter border (Figure 4).

### Spectral power and functional connectivity analysis of resting-state data

To obtain the source-based cortical activation, the distributed source model of the MEG data was estimated using the depth-weighted MNE analysis. The forward model of the MNE analysis was established using the overlapping sphere method, which presented each cortical vertex as a current dipole and included ~15,000 vertices [64]. Subsequently, the inverse operator, estimating the distribution of current sources that account for data recorded at the sensors, was calculated as follows: (1) the source orientations were constrained to be normal to the surface regions; (2) a depth weighting algorithm was used to compensate for any bias affecting the calculation of superficial sources; and (3) a regularization parameter, λ^2^ = 0.33, was used to minimize numerical instability, reduce the sensitivity of the MNE to noise, and effectively obtain a spatially smoothed solution. The regularization parameter determines the weight to be assigned to the MEG signal model relative to the background noise model; in other words, it is related to the signal to noise ratio (SNR). Therefore, it is defined as the reciprocal of the SNR of the MEG recordings. In the Brainstorm software, the default SNR is "3", which adopts the definition of SNR from the original MNE software [65]. The cortical source model of each participant was then morphed into a common source space defined by ICBM152 anatomy. The MNE analysis was performed using the Brainstorm program [66].

**Figure 5:**
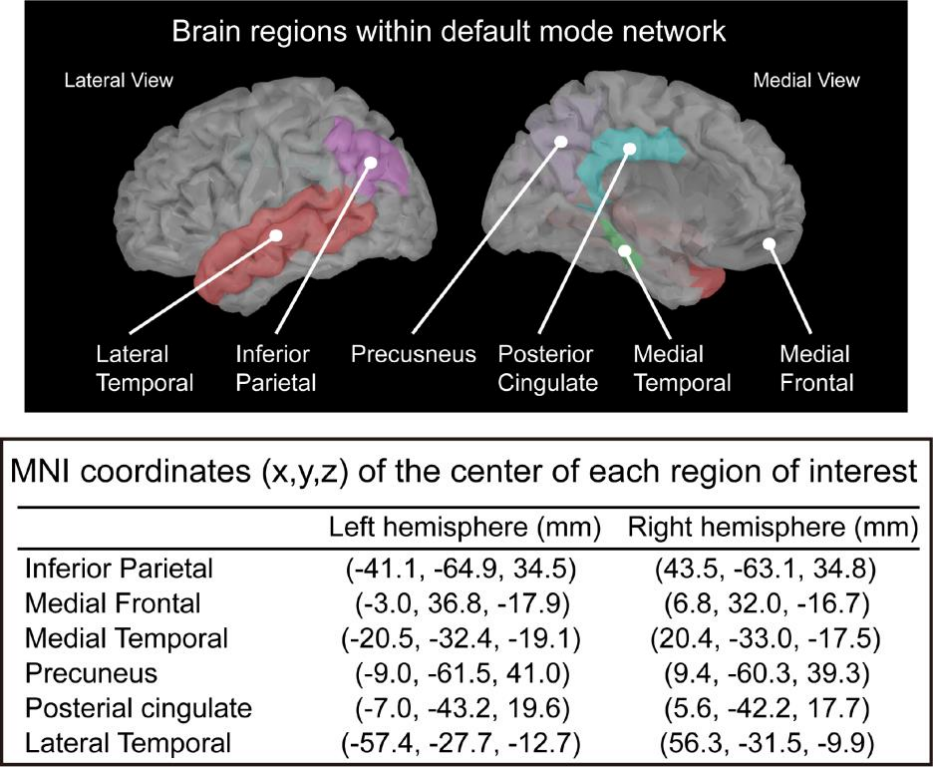
**Selected regions of interest within the default mode network and the corresponding MNI coordinates.**

In this study, we defined the ROIs in the T1 template volume using Mindboggle cortical parcellation [67]. We selected 12 DMN-related brain regions based on previous studies [23, 68–71], including the bilateral PCC, PCu, inferior parietal lobule, medial temporal cortex, medial frontal cortex, and LTC (Figure 5). In the MEG source analysis, each cortical vertex (~15,000 vertices in the cortex) represents a current dipole. The averaged current density from all vertices within the ROI was obtained to further estimate the source-based oscillatory power and functional connectivity [63, 68, 69, 72].

The dynamic source density in each ROI was further analyzed using the Brainstorm software to extract the oscillatory characteristics and functional connectivity in the DMN. Firstly, the estimate of the power spectral density (PSD) on each ROI was calculated using the Welch method (window duration 5 s with 50% overlap). The spectral power was normalized by means of dividing the power at each frequency band by the total power, which has been reported to adequately reduce the inter-individual variability [73]. Secondly, amplitude envelope correlation (AEC) analysis was used to determine the oscillatory functional connectivity. The amplitude envelope is defined as the absolute value of the Hilbert transform of a given cortical oscillation, which is obtained from the bandpass-filtered cortical source activities at each frequency band, and reflects the amplitude fluctuations in an oscillation over time [74]. AEC is calculated by correlating the amplitude envelopes of the cortical oscillatory activities from two ROIs. High AEC values indicate synchronous amplitude envelope fluctuations between cortical areas or networks. The connectivity between 12 ROIs of the DMN constructed the full 12×12 adjacency matrix. The node strengths (the sum of AEC values connected to the node) of the 12 regions were individually estimated to represent the magnitude of connectivity within the network [68, 72]. Spectral power (i.e., PSD) and oscillatory connectivity (i.e., AEC) were categorized by frequency bands: delta (2–4 Hz), theta (5–7 Hz), alpha (8–12 Hz), beta (15–29 Hz), gamma1 (30–59 Hz), and gamma2 (60–90 Hz).

### Statistical analysis

The demographics and clinical profiles of the HC and SCD groups were compared using independent t- or chi-squared tests, as appropriate. The group differences in the spectral power and node strength of each ROI at each frequency band were examined using analysis of variance (ANOVA) with the correction for multiple comparisons using a false discovery rate (FDR). In the subsequent analysis, the AEC values between ROIs at specific frequency bands, with significant difference in node strength, were compared between groups. To control the type I errors detected by the multiple comparisons of AEC values, p < 0.0045 (i.e., 0.05/11) was considered statistically significant using the Bonferroni method. Furthermore, the network measurements with significant between-group differences were also evaluated to determine the diagnostic value of SCD using the ROC curve analysis, which provided the AUC for the evaluation of the performance. All data are presented as the mean ± standard deviation (SD).
